#  Monitoring and Treatment of Acute Kidney Injury in Children with Acute Lymphoblastic Leukemia After High Dose Methotrexate Chemotherapy

**Published:** 2016

**Authors:** Cong-Ping Wang

**Affiliations:** *Eye & Ent Hospital of Fudan University, Shanghai 200031, China*

**Keywords:** Acute kidney injury, Acute lymphoblastic leukemia, Methotrexate chemotherapy, Children

## Abstract

To investigate acute kidney injury (AKI) in children with acute lymphoblastic leukemia (ALL) who received high dose methotrexate (MTX) chemotherapy and explore the corresponding treatment. Methods 180 children who received high dose MTX chemotherapy were observed with serum MTX concentration and serum creatinine. Patients with AKI of stage 3 or poor response to conventional treatment were performed on hemodialysis and assessed the treatment outcome. Results 9 patients (5%) have appeared AKI, including 7 cases of AKI of stage 3. However, there were not any significant correlation between age, gender, serum MTX concentration and AKI, respectively. Compared with normal serum MTX concentration, the patients with high serum MTX concentration easily were developed to AKI, the MTX and serum creatinine concentration had been significantly decreased in 9 patients after hemodialysis. Conclusion AKI has appeared in some children with ALL who receive high dose MTX chemotherapy, and this may due to increase of serum MTX concentration. The monitoring of serum MTX concentration and AKI index could help to find out AKI, and even to prevent the occurrence of it. Furthermore, once AKI is present, those patients with AKI stage 3 or poor response to conventional treatment should be performed on hemodialysis treatment.

## Introduction

Acute lymphoblastic leukemia (ALL) is the most common blood tumor in the children. The treatment was developed quickly these years and 5-year survival rate can reach more than 90%. In which, high dose methotrexate (MTX) therapy play an important role in improving remission rate and prolong life when widely used as effective measures for shelter treatment and whole body consolidation treatment because it could overcome the shortcoming of conventional dose chemotherapeutics, which are hard to traverse blood-brain barrier and blood-testis barrier. However, with the increase of MTX dose, the related side effects increased simultaneously, such as mucosal damage, bone marrow suppression, hepatic and renal functions injuryˏ etc (1, 2).

In the past few years, the proposal of constant amendment on AKI diagnosis help greatly on the early detection and prevention of AKI (3, 4). In present, we found AKI timely through monitoring serum MTX concentration, 7-hydroxymethotrexate (7-OH-MTX)/MTX index and AKI index. Patients with AKI have be better after hemodialysis based on the fully hydration, alkalinizing and calcium folinat (CF) rescue. 

## Materials and Methods

*General information*

From Jan 2010 to Jan 2014, there were 180 acute lymphocytic leukemia (ALL) children treated in pediatric hematological department, in which, 68 cases were of low risk, 80 cases were of average risk, and 32 cases are of high risk. 122 cases (68 boys, 54 girls) from 17 months to 16 years old (average age: 6.4 years old) were in the induced remission stage, the other 58 cases (30 boys, 28 girls) from 2 to 13 years old (average age: 5.9 years old) were in the maintenance treatment stage. 

The diagnosis was according to the advice on diagnosis and treatment of children with ALL (the third edition) released by pediatric blood disease group of Chinese Medical Association in 2006 (5). This research was approved by soochow university affiliated children’s hospital ethics committee and was agreed by parents of the patient.

*High dose MTX Treatment Protocols*

According to the Chinese children leukemia group - ALL 2008 solutions group (6). High dose-MTX chemotherapy include four course of treatment. Total MTX dose is 3-5g/m^2^, 1/10 total dose, as flushing dose, was rapidly dripped intravenously in 30 min, then the remainder dose was infused in a steady speed in the next 23.5 h. Combined injections of MTX+Dexamethasone +ARA-C were performed 2 h after the flushing injection of MTX. CF rescue were performed 36 h after the MTX chemotherapy, with CF dose of 15 mg/m^2^, each time (3 times, every 6 h).

*Measurement of MTX blood drug concentration and CF rescue*

3 mL blood samples were collected at the 24, 48, 72 h after MTX infusion, HPLC method was applied to measure the serum MTX concentration and metabolite 7-OH-MTX concentration. The serum samples were treated with perchloric acid for sedimentation and high speed centrifugation, then the supernatant was taken for analysis. Chromatographic separation was performed on Synergi-4u-Fusion-RP 80A analytical column with mobile phase consisted of phosphate buffer (pH 6.8)-methanol (78:22) at a flow rate of 0.8 mL·min^-1^. The UV detection wavelength was set at 306 nm and the column temperature was 30 ℃. If the MTX concentration at 24 h is over 10 umol/L, over 1.0 umol/L at 48 h, or over 0.1 umol/L at 72 h, it indicates that MTX metabolism is slow and the blood concentration is high, which need to CF rescue. The serum MTX concentration which is less than the above level at each check time were seen as normal. Monitoring serum MTX concentration every 24 h and stopping rescue until the MTX concentration decreased than 0.1 umol/L.

**Table 1:** KDIGO classification

Stage	Serum creatinine criteria	Urine output criteria
1	1.5 – 1.9 times baseline OR ≥ 0.3mg/dL in ≤ 48h	<0.5mL/kg/h for 6 – 12h
2	2 – 2.9 times baseline	<0.5mL/kg/h for ≥ 12h
3	≥ 3 times baseline OR increase in SCr to ≥ 4.0mg/dL OR initiation of RRT	<0.3mL/kg/h for≥ 24h OR anuria for ≥ 12h

note: RRT(renal replacement treatment)

**Table 2:** AKI stage and occurrence time

Patient number	AKI stage	AKI occurrence time
1	3	<48h
2	3	<48h
3	2	>48h
4	2	>48h
5	3	>48h
6	3	>48h
7	3	>48h
8	3	>48h
9	3	>48h

**Table 3:** Relationship between MTX blood concentration and AKI

MTX blood concentration	AKI Occurred	AKI Not Occurred
normal MTX	0	168
high MTX	9	3

**Table 4:** Relationship between 7-OHMTX/MTX index and AKI occurred after 48h

MTX blood concentration	AKI Occurred	AKI Not Occurred
7-OH-MTX/MTX＜2	7	50
7-OH-MTX/MTX＞2	2	130

**Table 5:** Index change ( before and after hemodialysis) in children with HD-MTX induced AKI

Index	Before hemodialysis	After hemodialysis
Serum creatinine (umol/L)	191.164±63.51	62.19±26.34*
MTX blood level (umol/L)	15.068 ±11.32	0.21± 0.11*

* P<0.05 vs before hemodialysis

*Monitoring and treatment of AKI*

We use KIDGO guideline for AKI to diagnose and classify AKI (Table 1) (4). To ensure the exactness of the diagnosis and classification of AKI, we detect the basic kidney function before high dose MTX treatment, then continue monitoring it and the urine volume throughout treatment. 

The CF rescue was added when AKI occurred. Dialyser is fresenius F5 type hollow fiber dialyser (area of 1.0 m^2^) and the corresponding line. Vascular access adopts 8 F or 11.5 F single double lumen tube femoral venous indwelling needle. Dialysate is bicarbonate solution, which all patients need to puncture catheter through the femoral vein, after taking blood out, it through the Pre-Pierce Dialyser and piping. Heparin: starting dose 0.5 mg/Kg, maintain 0.25 mg/Kg, during dialysis, stop taking at 30 min before the end of dialysis. Dialysate flow rate was 500 Ml/min, blood flow rate was 3-5 mL/Kg. Once every other day, 2-4 h each time, 2-5 treatments made up one course.

*Statistical method*

Data were analyzed by SPSS 19.0 software using comparative t-test and chi-square test (Mean ± SD). A *P* value less than 0.05 was considered as statistically significance. 

## Results

*AKI occurrence rate*

AKI appeared at 9 patients (5%), including 7 cases of AKI stage 3, however, there were not any significant correlation between age, gender, MTX concentration and AKI, 2 cases occurred within 48 h, the other 7 cases occurred after 48 h. (Table 2.)

*Relationship between MTX concentration and AKI*

Compared with normal serum MTX concentration, the patients with high serum MTX concentration easily were developed to AKI. (Table 3. P<0.05).

*Relationship between 7-OH-MTX/MTX index and AKI after 48 h*

According to the 7-OH-MTX/MTX index (<2 or >2), 180 cases without AKI after 48 h were divided into two groups, and then the AKI occurrence rate were compared. The result showed that the AKI occurrence rate after 48h (7-OHMTX/MTX<2) increased obviously (Table 4.).

*The effect of hematodialysis on AKI induced by HD-MTX*

9 children had been received hemodialysis (7 cases with AKI stage 3, 2 case with AKI stage 2 and poor response to conventional treatment). The MTX and serum creatinine concentration of 9 patients had significantly decreased after hemodialysis. (Table 5.)

## Discussion

MTX is one kind of anti-folic acid metabolite, mainly affect the S phase of cell cycle. The structure of MTX is similar to folic acid and has high affinity with dihydrofolate reductase. As a dihydrofolate reductase inhibitor, which can hinder dihydrofolic acid into leucovorin and the DNA synthesis, therefore, play the role of killing tumor cells (7). In children, high dose-MTX treatment can maintain high concentration in long time which play an important role in the prevention of those “shelter area”, such as central nervous system and testis. But when used in high dose, MTX has obvious toxicity. MTX mainly work on fast-growing and fast-updated cells, therefore skin mucous membrane damage and myelosuppression are common adverse reactions. After the large doses administration, 90% MTX is excreted by kidney, which kidney injury will also inevitable. Therefore, high MTX concentration may cause severe adverse reaction such as bone marrow suppression and aggravate renal injury, even threaten the patient›s life (8).

This research found that in high dose MTX treatment, 5% children occurred AKI. In general, the possible mechanism of MTX induced AKI are the following: 1.) allergic reaction: usually present as interstitial nephritis; 2.) renal tubule toxicity: crystals formation of 7-OH-MTX in acid condition. The formed crystals have deposited in renal tubular to cause the injury of renal tubular function. 3.) Glomerular injury: contracting of afferent glomerular arteriole and renal hypoperfusion. Among the three aspects, renal tubule toxicity may be the main factor (9, 10). 

In the high dose MTX treatment, full hydration can increase urine volume and MTX excretion, and then reduce serum MTX concentration. Full alkalization can lower the crystal formation of MTX and kidney injury in renal tubule. All these factors can obviously decrease the occurrence rate of AKI. But because of the individual different for MTX concentration, patients have a various serum MTX concentration. Our research found that the AKI occurrence of children is accompanied with high serum MTX concentration. Therefore, serum MTX concentration and AKI index must be monitored in order to early identify it.

In this research, 7 patients occurred AKI after 48 h. For MTX concentration, serum MTX concentration after 48 h of 4 cases was lower than 1.0 umol/L, but MTX serum concentration after 72 h exceed 0.1umol/L, indicating MTX clearance has been delayed. Furthermore, side-effect of MTX was closely related to the elimination rate in later stage. The faster the elimination was, the smaller the cumulative toxicity effect appeared in the later stage. To predict the elimination rate in later stage and carry out rescue treatment can catch the best rescue chance to prevent the development of AKI. Therefore, we consider that patients with serum MTX<1μmol·L^-1^ and 7-OH-MTX/MTX<2 should be paid great attention to adverse reaction induced by MTX in the later stage. We could increase CF rescue dose to make early intervention and decrease the occurrence of AKI. Once AKI occurred, besides adequate hydration and alkalization by CF rescue, we also perform hemodialysis actively on the children with high risk of AKI. The result showed that serum creatinine of patients with AKI stage 3 decreased obviously after hemodialysis accompanied the decrease of serum MTX concentration. It means that the hemodialysis is greatly helpful to the AKI patients. 

In our study, there are three points need to be paid attention: 1.) to monitor MTX blood concentration and AKI diagnosis index, especially MTX blood concentration after 48 h. We need to adjust CF rescue dose and times based on serum MTX concentration until achieving non-toxic concentration. In addition to, the early monitoring of 7-OH-MTX/MTX index can help to estimate MTX clearance rate in the later stage and decrease the occurrence rate of AKI. 2.) The full hydration and alkalization (urine PH>7.0 in urine) is important in high dose MTX treatment. 3.) once AKI is present, the children with AKI stage 3 or poor response to conventional treatment should be performed on hemodialysis actively as early as possible.
